# Association between human blood metabolome and the risk of hypertension

**DOI:** 10.1186/s12863-023-01180-z

**Published:** 2023-12-15

**Authors:** Nannan Dai, Yujuan Deng, Baishi Wang

**Affiliations:** 1 The Eco-city Hospital of Tianjin Fifth Central Hospital, Tianjin, 300467 China; 2 School of Mathematical Sciences, Hebei Normal University, Shijiazhuang, 050010 China; 3 College of Future Information Technology, Shijiazhuang University, Shijiazhuang, 050035 China

**Keywords:** Hypertension, Diastolic blood pressure, Systolic blood pressure, Mendelian randomization

## Abstract

Hypertension, commonly referred to as high blood pressure, is a chronic medical condition characterized by persistently elevated blood pressure levels. It is a prevalent global health issue, affecting a significant portion of the population worldwide. Hypertension is often asymptomatic, making it a silent but potentially dangerous condition if left untreated. Genetic instruments for 1,091 were from a recent comprehensive metabolome genome-wide association study (GWAS). Summary statistics of diastolic blood pressure (DBP) and systolic blood pressure (SBP) involving 757,601 sample size were analyzed. Two-sample Mendelian Randomization (MR) was conducted to assess causal effect of metabolites on DBP and SBP risk, and reverse MR analysis was performed to identify the DBP/SBP causal effect on blood metabolites. Twelve and twenty-two metabolites were identified to be associated with DBP and SBP, respectively. Sensitive analysis showed four metabolites had robustness association on BP. Reverse MR demonstrated DBP and SBP could decrease the tricosanoyl sphingomyelin (d18:1/23:0)* level and increase the 2-hydroxyhippurate (salicylurate) level in blood, respectively. Our findings reveal an association between blood metabolites and blood pressure (DBP and SBP), suggesting potential therapeutic targets for hypertension intervention.

**Supplementary Information:**

The online version contains supplementary material available at 10.1186/s12863-023-01180-z.

## Introduction

Hypertension is a chronic disease in which arterial blood pressure increases continuously and involves a variety of cells or organs [1]. Hypertension has no typical symptoms in its early stages and is often overlooked. It is estimated that 1.28 billion adults aged 30–79 years worldwide have hypertension, and less than half of adults with hypertension have been diagnosed and treated [2]. As the disease progresses to the clinical stage, it can cause serious damage to the heart, brain, kidneys, and other organs, making it one of the main contributors to premature death. Therefore, it is of great significance to understand the pathogenesis and new prevention targets of hypertension.

Metabolites in the blood are important substances that reflect the biological physiological state. As genes and the environment change, the concentration of metabolites will also change, resulting in individual differences [3]. Increasing research supports the notion that hypertension is a metabolic disease [4]. Studies conducted on hypertensive animal models have demonstrated alterations in specific metabolites found in the serum and urine of hypertensive mice [5]. Yanan et al. comprehensively analyzed metabolic changes occurring at different stages of hypertension in a rat model [6]. However, although these association studies have provided valuable insights, confirming the exact relationship between hypertension and metabolites in humans is challenging due to confounding factors and reverse causality. To address this, our study aims to employ a two-sample Mendelian randomization analysis to evaluate the potential causal relationship between blood metabolites and the risk of hypertension in human. By utilizing genetic variations as instrumental variables, this approach explores the causal link between exposure (blood metabolites) and the outcome (hypertension). We conducted a Mendelian randomization analysis utilizing the latest comprehensive genome-wide association study (GWAS) summary statistics of blood metabolites to assess their causal relationship with diastolic and systolic blood pressure, providing insights into potential therapeutic targets for intervening in hypertension.

## Materials and methods

### Study design

**Fig. 1:**
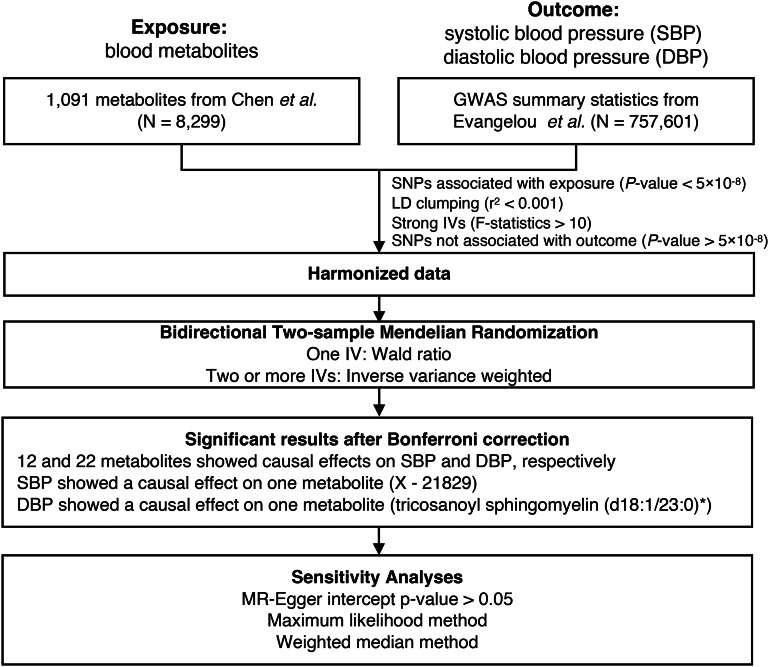
Overview of this MR study

The study design is depicted in Fig. 1, providing an overview of the current study. The methods employed and the characteristics of the study participants are elaborated upon in the following sections.

Genetic variants linked to metabolite levels were obtained from a comprehensive genome-wide association study (GWAS) conducted on the Canadian Longitudinal Study on Aging (CLSA) cohort. These genetic variants were then employed as instrumental variables (IVs) to assess the relationship between metabolite levels and hypertension traits.

### Data source for blood metabolome and hypertension

A recent extensive genome-wide association study (GWAS) from Canadian Longitudinal Study on Aging (CLSA) cohort focused on investigating the relationship between single nucleotide polymorphisms (SNPs) and the human metabolome. In the study [7], researchers analyzed data on 1,091 metabolic traits (Table S1), examining a total of 8,299 participants and approximately 15.4 million SNPs. By extracting summary-level information from this study, valuable insights into the associations between genetic variations and the human metabolome were obtained for instrumental variables (IVs).

The summary statistics for GWAS of the diastolic blood pressure (DBP) and systolic blood pressure (SBP) were obtained from Evangelou et al. study, including UK Biobank (UKB) genome-wide association study (GWAS) and International Consortium for Blood Pressure (ICBP) GWAS. The UKB data involved in a total of 458,577 UKB participants genotyped using a custom Affymetrix UK Biobank Axiom Array chip or custom Affymetrix UK BiLEVE Axiom Array chip. The ICBP GWAS comprises a comprehensive collection of 77 individual studies, including Age, gene/Enviroment Susceptibility-Reykjavik Study (AGES), Atherosclerosis Risk in Communities (ARIC), Austrian Stroke Prevention Study (ASPS), British 1958 birth cohort (B58C), Busselton Health Study (BHS), Cardiovascular Health Study (CHS), Cohorte Lausannoise (COLAUS), Genetic Predisposition of Coronary Heart Disease in Patients Verified with Coronary Angiogram (controls for this study are a part of the National FINRISK Studies) (COROGENE), CROATIA-Korcula (CROATIA-Korcula), CROATIA-Split (CROATIA-Split), CROATIA-Vis (CROATIA-Vis), Estonian Genome Center (EGCUT), Estonian Genome Center (EGCUT2), European Prospective Investigation in Cancer and Nutrition (EPIC), Erasmus Rucphen Family (ERF), Fenland Study (Fenland), Framingham Heart Study (FHS), Predicting CVD in FINRISK cohorts, cases (FINNRISK CASE), Predicting CVD in FINRISK cohorts, controls (FINRISK_ctrl), Finland-United States Investigation of NIDDM Genetics Study (FUSION), Genetic Regulation of Ambulatory Blood Pressure in the Community (GRAPHIC), Health 2000 controls (H2000), The Health Aging and Body Composition Study (Health ABC), HTO, Italian Network of Genetic Isolates - Val Borbera (INGI_VB), Genetic Park of Cilento and Vallo di Diano Project (Cilento study), Italian Network on Genetic Isolates - Friuli Venezia Giulia Genetic Park (INGI-FVG), Italian Network on Genetic Isolates - Carlantino Project (INIG-CARL), Charles R. Bronfman Institute for Personalized Medicine (IPM) BioBank Genome Wide Association Study of Cardiovascular, Renal and Metabolic Phenotypes (IPM), Kooperative Gesundheitsforschung in der Region Augsburg (KORAS3), Cooperative Health Research in the Region of Augsburg (Survey 4) (KORAS4), Lothian Birth Cohort 1921 (LBC1921), Lothian Birth Cohort 1936 (LBC1921), London Life Sciences Prospective Population Study (LOLIPOP_EW610), Multi-Ethnic Study of Atherosclerosis (MESA), MICROS (MICROS), Myocardial Infarction Genetics Consortium (MIGen), Netherlands Study of Depression and Anxiety (NESDA), The Northern Sweden Population Health Study (NSPHS), Netherlands Twin Register (NTR), Orkney Complex Disease Study (ORCADES), PHArmacogenetic Study of Statins in the Elderly at risk (PROSPER), Prospective Investigation of the Vasculature in Uppsala Seniors (PIVUS), Precocious Coronary Artery Disease (PROCARDIS), Rotterdam Study 1 (RSI), Rotterdam Study 2 (RSII), Rotterdam Study 3 (RSIII), Study of Health in Pomerania (SHIP), Swedish Twin Register (STR), Tracking Adolescents’ Individual Live Surveys (TRAILS), Tracking Adolescents’ Individual Live Surveys - Clinical Cohort (TRAILS-CC), Uppsala Longitudinal Study of Adult Men (ULSAM), Women’s Genome Health Study (WGHS), The Young Finns Study (YFS), Anglo-Scandinavian Cardiac Outcomes Trial (Scandinavia) (ASCOT-SC), Anglo-Scandinavian Cardiac Outcomes Trial (UK/Ireland) (ASCOT-UK), The British Genetics of Hypertension Trial (BRIGHT), The Three City Study - Dijon (3 C-DIJON), European Prospective Investigation into Cancer and Nutrition - cardiovascular disease study (EPIC-CVD), The Fenland Study (Fenland (Fenland-GWAS)), The Fenland Study (Fenland (Fenland-OMICS)), European Prospective Investigation into Cancer - InterAct Study (EPIC-InterAct (EPIC-InterAct-GWAS)), European Prospective Investigation into Cancer - Norfolk Cohort (EPIC-Norfolk), Genetic and phenotypic determinants of blood pressure and other cardiovascular risk factors (GAPP), Genetics of Diabetes and Audit Research Tayside Study (GoDARTS), Generation Scotland (GS:SFHS), Hunter Community Study (HCS), Justification for the Use of Statin in Prevention: An Intervention Trial Evaluating Rosuvastatin (JUPITER), The Lifelines Cohort (Lifelines), Malmö Diet and Cancer study (MDC), Metabolic Syndrome in Men (METSIM), Netherlands Epidemiology of Obesity Study (NEO), Prevention of REnal and Vascular ENd-stage Disease (PREVEND), SardiNIA (SardiNIA), TwinsUK (TWINSUK), United Kingdom Household Longitudinal Study (UKHLS), collectively involving a substantial participant pool of 299,024 individuals. All the participants in the ICBP GWAS are European, and the genotype were imputed using either the 1000 Genomes Project Phase 1 integrated release v.3 (March 2012) all-ancestry reference panel or the Haplotype Reference Consortium (HRC) panel.

### Genetic instruments for blood metabolites

To identify genetic instruments for Mendelian randomization (MR) analysis, SNPs associated with blood metabolites at the genome-wide significant threshold (*p*-value < 5 × 10^− 8^) were utilized as instrumental variables (IVs) in this study [8–10]. The selection procedure for these instruments is depicted in Fig. 1 and involved the following steps: (1) SNPs underwent LD clumping, considering a threshold of r^2^ < 0.001 within a 10,000 kb window. This process accounted for potential linkage disequilibrium between SNPs, which could otherwise lead to an overestimation of instrumental strength and overly precise effect estimates [11]. (2) Only SNPs with matching alleles between the exposure and outcome datasets were retained; (3) Strict criteria were applied to include only robust IVs, requiring an *F-statistic* ≥ 10; (4) The IVs selected for the exposure variable were ensured to have no direct association with the outcome, with a *p*-value > 5 × 10^− 8^.

### MR analyses for likely causally associated metabolites for DBP and SBP risk

Two-sample MR was utilized to estimate the associations between genetically predicted metabolite levels and DBP and SBP risk, respectively, following the recommendations of the STROBE-MR statement [12]. In the current study, the genetic instruments for each metabolite of interest are determined from the CLSA study, by focusing on SNPs associated with levels of each metabolite of interest at *p*-value < 5 × 10^− 8^. One key benefit of conducting Mendelian randomization (MR) analysis is that it solely relies on GWAS summary statistics, eliminating the need for individual-level data. Two-sample Mendelian randomization (MR) offers several advantages, including increased statistical power, mitigation of bias stemming from unmeasured confounders or reverse causality, and enhanced flexibility when selecting exposure and outcome variables [13].

After extracting the association estimates between the variants and the exposures or the outcome, the Wald Ratio (WR, when only one IV exists) or inverse variance weighting (IVW, when two or more IVs are available) method was used to conduct main MR analysis [8, 14–16]. Heterogeneity among genetic instruments was assessed using Cochran’s Q test. The significance of the MR results was determined using Bonferroni correction of 0.05 (*p*-value = 7.05 × 10^− 5^ = 0.05/707 for DBP and 7.11 × 10^− 5^ = 0.05/703 for SBP). All the MR analysis was performed using TwoSampleMR R package [17].

To validate the robustness of our results in the face of possible violations of the MR assumptions, we conducted two sensitivity analyses when two or more IVs were available: (1) We used MR-Egger’s intercept to estimate the possibility of horizontal pleiotropy [18]; (2) We employed the maximum likelihood method and the weighted median-based method to assess the significant of relationships between metabolites and hypertension traits.

### Reverse MR analyses

We used the same parameters (*p*-value < 5 × 10^− 8^) to extract SNPs from DBP and SBP from GWAS summary statistics. Then, we selected independent SNPs (r^2^ < 0.001 in the European panel using Two Sample MR software) used these as IVs in an MR analysis on all the 1,091 metabolite traits. Similarity, Bonferroni correction of 0.05 (*p*-value = 2.29 × 10^− 5^ = 0.05/(1091 + 1091)) was used as the threshold to identify significant association results.

## Results

### Levels of blood metabolites associate with DBP risk

**Fig. 2:**
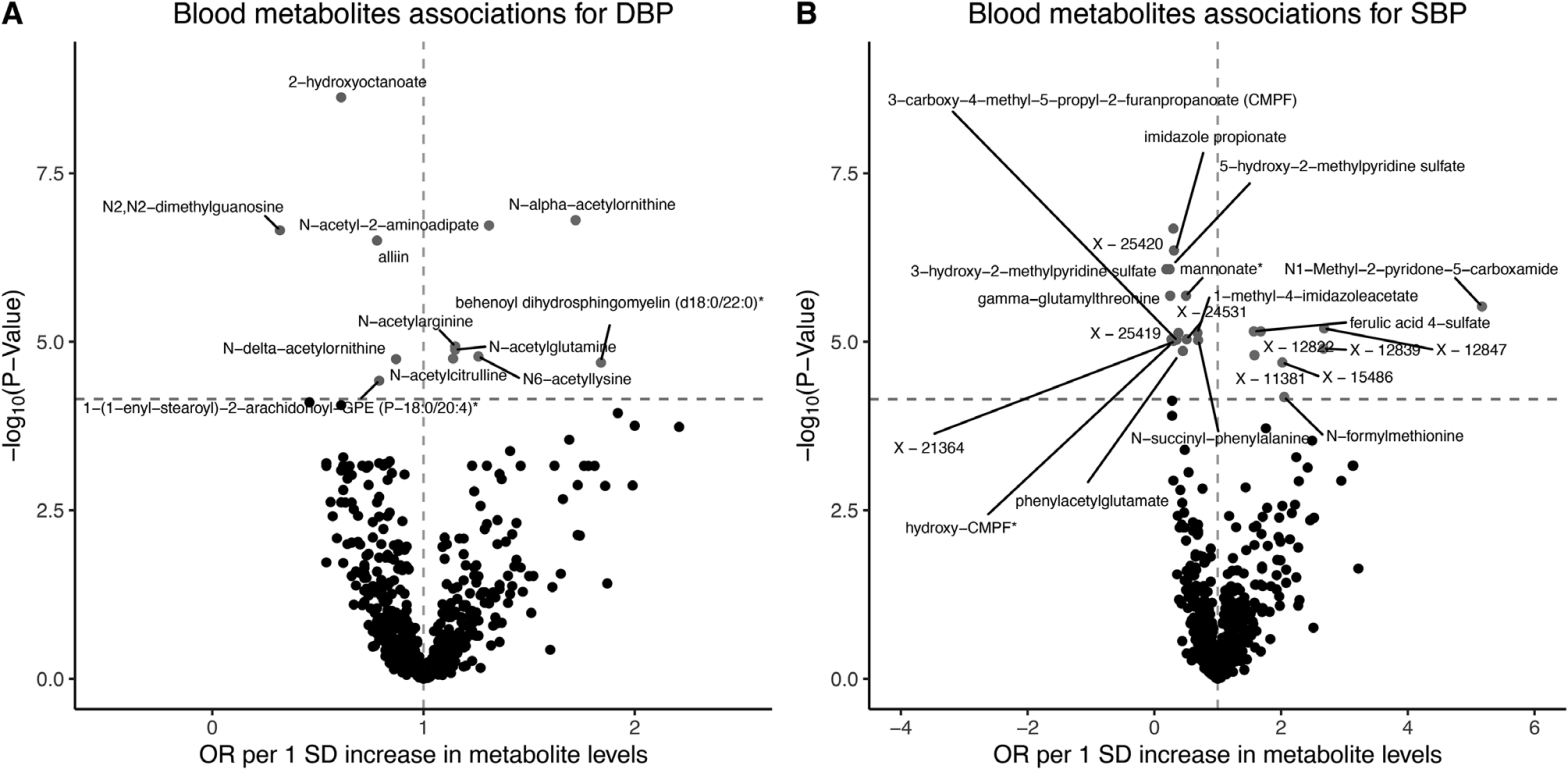
Blood metabolites associations for hypertension. (**A**) Volcano plot demonstrating negative (blue) and positive (red) blood metabolites associations with DBP; points are colored where P < 0.05/707. (**B**) Volcano plot demonstrating negative (blue) and positive (red) blood metabolites associations with SBP; points are colored where P < 0.05/703

According to the Method section, a total of 707 blood metabolites were selected based on the mentioned strategy for further analysis. Causal relationships between these 707 blood metabolites and DBP were examined using the WR or IVW method. Following Bonferroni correction for multiple testing, a total of 12 blood metabolites were found to be significantly associated with DBP. Among these metabolites, 7 (N-alpha-acetylornithine, N-acetyl-2-aminoadipate, N-acetylarginine, N-acetylglutamine, N6-acetyllysine, N-acetylcitrulline, and behenoyl dihydrosphingomyelin [d18:0/22:0]) demonstrated a positive effect on DBP, while 5 metabolites (2-hydroxyoctanoate, N2,N2-dimethylguanosine, alliin, N-delta-acetylornithine, and 1-(1-enyl-stearoyl)-2-arachidonoyl-GPE (P-18:0/20:4)*) showed a negative effect on DBP (Fig. 2A and Table S2). The IVs employed for the MR analysis, specifically derived from the 12 metabolites that demonstrated significant associations, can be found in Table S3.

In the sensitivity analysis, seven metabolites were assessed, namely 1-(1-enyl-stearoyl)-2-arachidonoyl-GPE (P-18:0/20:4)*, 2-hydroxyoctanoate, behenoyl dihydrosphingomyelin (d18:0/22:0)*, N-acetylarginine, N-acetylcitrulline, N-acetylglutamine, and N-delta-acetylornithine, which had two or more than two IVs. For behenoyl dihydrosphingomyelin (d18:0/22:0)*, both the Maximum Likelihood (*p*-value = 1.05 × 10^− 8^) and Weighted Median (*p*-value = 7.13 × 10^− 7^) methods revealed a significant association between the metabolite and SBP. The *p*-value from the Egger intercept analysis was 0.28, indicating no evidence of pleiotropy affecting the instrumental variables. Heterogeneity tests were conducted to assess potential heterogeneity among the instrumental variable estimates, and the results indicated that there was no significant heterogeneity present (*p*-value = 0.64). Similar findings were observed for the metabolites 1-(1-enyl-stearoyl)-2-arachidonoyl-GPE (P-18:0/20:4)*, 2-hydroxyoctanoate, N-acetylarginine, N-acetylcitrulline, N-acetylglutamine, and N-delta-acetylornithine (Table S4).

### Levels of blood metabolites associate with SBP risk

In the present study, a total of 703 blood metabolites were identified to possess instrumental IVs for MR analysis. The WR or IVW method was utilized to investigate the causal relationships between these 703 blood metabolites and SBP. Upon applying the Bonferroni correction for multiple testing, a total of 22 blood metabolites were found to exhibit significant associations with SBP. Among these metabolites, 8 namely N1-Methyl-2-pyridone-5-carboxamide, X-12847, X-12822, ferulic acid 4-sulfate, X-12839, X-11381, N-formylmethionine, and X-15486 displayed a positive effect on SBP. Conversely, 14 metabolites including X-25420, imidazole propionate, 5-hydroxy-2-methylpyridine sulfate, 3-hydroxy-2-methylpyridine sulfate, gamma-glutamylthreonine, mannonate*, 1-methyl-4-imidazoleacetate, X-25419, 3-carboxy-4-methyl-5-propyl-2-furanpropanoate (CMPF), X-24531, X-21364, N-succinyl-phenylalanine, hydroxy-CMPF*, and phenylacetylglutamate were found to exert a negative effect on SBP (Fig. 2B and Table S5). Detailed information on the IVs used for the MR analysis derived from the 22 significant metabolites associated with blood pressure can be found in Table S6.

In the sensitivity analysis, only one metabolite (X-11381) with four instrumental variables IVs was further examined. The results demonstrated a significant association between X-11381 and SBP using both the Maximum Likelihood (*p*-value = 2.46 × 10^− 5^) and Weighted Median (*p*-value = 5.17 × 10^− 5^) methods. Furthermore, no significant heterogeneity (*p*-value = 0.67) or pleiotropy (*p*-value = 0.42) was detected among the instrumental variables used in the analysis. These findings indicate that the association between X-11381 and SBP is consistent across the different IVs, and there is no evidence of substantial heterogeneity or pleiotropy affecting the results (Table S7).

### Comparison of the associated metabolites between DBP and SBP

In the current study, we didn’t identify any metabolites significantly associated with DBP and SBP simultaneously. Out of the 12 metabolites significantly associated with DBP, only one metabolite, namely behenoyl dihydrosphingomyelin (d18:0/22:0)*, exhibited a positive association with SBP at a *p*-value below 0.05 (Table S2 and Table S5).

Conversely, we further investigated whether the 22 metabolites significantly associated with SBP demonstrated potential associations with DBP. Among these metabolites, instrumental variables (IVs) were available for 19 metabolites, excluding 5-hydroxy-2-methylpyridine sulfate, 3-hydroxy-2-methylpyridine sulfate, and N1-Methyl-2-pyridone-5-carboxamide. Interestingly, all the metabolites showed potential associations with DBP, displaying the same effect direction, and achieving statistical significance at a *p*-value below 0.05 (Tables S2 and S5).

### Reverse MR analysis assessing the effect of BP on blood metabolites

**Fig. 3:**
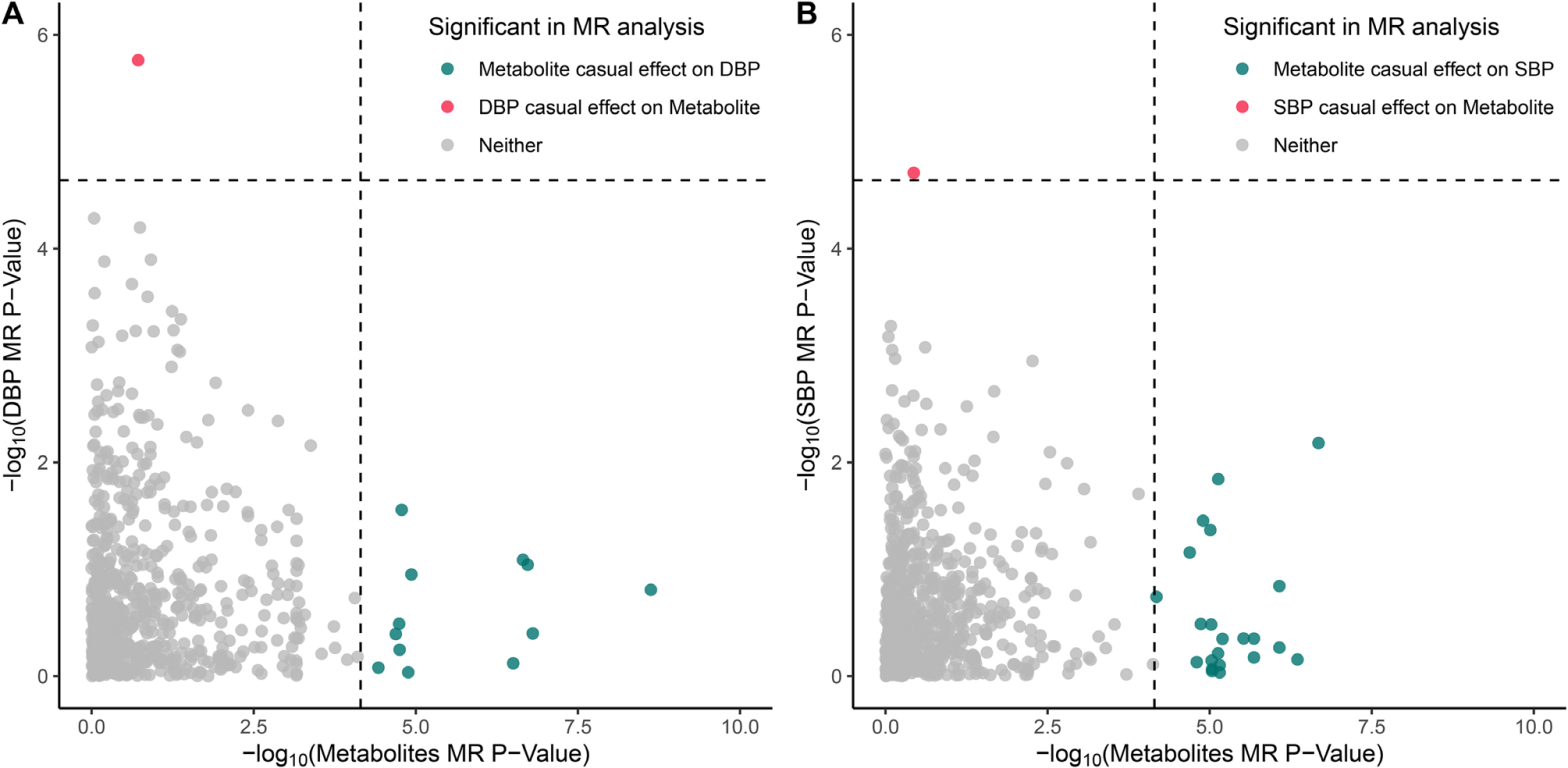
Bidirectional Mendelian randomization analysis supports causal associations for blood metabolites on hypertension and vice versa. (**A**) Comparison of bidirectional two-sample MR -log_10_ (*p*-values) for the 707 metabolites that could be investigated in both directions, where the x-axis indicates the *p*-value for a causal effect of blood metabolites on DBP and the y-axis indicates the *p*-value for a causal effect of DBP on blood metabolites. (**B**) Comparison of bidirectional two-sample MR -log_10_ (*p*-values) for the 703 metabolites that could be investigated in both directions, where the x-axis indicates the *p*-value for a causal effect of blood metabolites on SBP and the y-axis indicates the *p*-value for a causal effect of SBP on blood metabolites. Significant metabolites as indicated in the legend, while the dashed lines indicate significant threshold after Bonferroni correction

To further evaluate the causal impact of blood pressure (DBP and SBP) on blood metabolites, a reverse MR analysis was performed. The IVs were extracted from the GWAS summary for both DBP and SBP, as outlined in the Method section. The results revealed that DBP exhibited a significant association with only one metabolite, namely tricosanoyl sphingomyelin (d18:1/23:0)* (OR per SD increase: 0.98; 95% CI: 0.97–0.99; *p*-value = 1.73 × 10^− 6^) (Fig. 3 and Table S8). Similarly, SBP demonstrated a significant association with only one metabolite, specifically 2-hydroxyhippurate (salicylurate) (OR per SD increase: 1.01; 95% CI: 1.01–1.02; *p*-value = 1.69 × 10^− 5^) (Fig. 3 and Table S9).

## Discussion

In this study, a comprehensive two-sample MR approach was employed to examine the causal relationship between blood metabolite levels and blood pressure (DBP and SBP). A total of 12 metabolites were found to be significantly associated with DBP, while 22 metabolites showed a significant association with SBP. In reverse MR analysis, we identified DBP has negatively causal effect on tricosanoyl sphingomyelin (d18:1/23:0)* level and SBP has positive causal effect on 2-hydroxyhippurate (salicylurate) level.

To enhance the rigor of our selection process for instrumental variables (IVs), we employed more stringent criteria compared to a previous study [19]. However, for metabolites with multiple IVs (more than two), several sensitivity analyses were performed to validate the robustness of our findings. Specifically, four metabolites (1-(1-enyl-stearoyl)-2-arachidonoyl-GPE (P-18:0/20:4)*, behenoyl dihydrosphingomyelin [d18:0/22:0]*, N-acetylarginine, and N-acetylglutamine) were examined for their itssignificant association with DBP, and one metabolite (X-11,381) was investigated for its significant association with SBP. All sensitivity analyses consistently supported our main analysis results, as no significant pleiotropy or heterogeneity was observed in the study, indicating that the four metabolites under investigation are robustly associated with blood pressure. For the remaining results (8 metabolites on DBP, and 21 metabolites on SBP), we were unable to perform sensitivity analyses for these associations due to the limited number of available IVs. Therefore, further investigations with larger sample sizes are necessary to validate our findings.

In the previous Mendelian randomization (MR) analysis from Qiao et al. [19], three metabolites, namely N-acetylglycine (OR = 0.95, 95%CI: 0.92–0.97, FDR = 0.023), X-09026 (OR = 0.85, 95%CI: 0.78–0.92, FDR = 0.02), and X-14473 (OR = 0.94, 95%CI: 0.91–0.97, FDR = 0.04), were found to be significantly associated with DBP. However, no metabolites were identified as significantly associated with SBP. In the current study, we employed more stringent criteria in selecting IVs. We employed a threshold of 5 × 10^− 8^ for selecting significant SNPs whereas the prior study utilized 1 × 10^− 5^. We performed LD clumping using the default parameter (r^2^ < 0.001 within a 10,000 kb window) using the TwoSampleMR software. In contrast, the previous study used a more relaxed threshold with r^2^ < 0.1 and a physical distance of 500 kb. Additionally, we excluded weak IVs with *F-statistic* < 10 and SNPs directly significantly associated with outcome (*p*-value < 5 × 10^− 8^). Thus, in our study, most metabolites remained only one IV for further analysis. Furthermore, we used the latest metabolites GWAS summary with a larger sample size (8,299 verse 7,828) and a broader spectrum of metabolites (1,091 vs. 486), resulting in a more comprehensive and rigorous result. In our study, a causal effect analysis was performed between the metabolite N-acetylglycine and DBP. However, our results indicated that N-acetylglycine did not demonstrate a causal effect on DBP (OR = 1.13, 95%CI: 0.84–1.52, *p*-value = 0.42). These findings suggested that further investigations were necessary to validate the causal effect of N-acetylglycine on DBP. We cannot compare the other metabolites due to the lack of IVs in the current study. The glycine was reported genetically associated with lower CHD risk and found that this may be partly driven by blood pressure [20]. In our study, the glycine showed a negative causal effect on DBP (OR = 0.76, 95%CI: 0.63–0.92, *p*-value = 4.71 × 10^− 3^) and SBP (OR = 0.43, 95%CI: 0.24–0.78, *p*-value = 5.16 × 10^− 3^), which is consistent with our results.

Several limitations exist in our study. Firstly, despite utilizing a recent and comprehensive metabolite GWAS dataset, the sample size employed in our MR analysis was relatively small (N = 8,299). We used a more stringent threshold in selecting IVs, this approach significantly improved the selection of strong IVs, but it also resulted in a limited number of IVs explaining only a small portion of the variance. It is imperative to validate our current results using a larger sample size and additional GWAS summaries of metabolites. Secondly, our study primarily focused on individuals of European ancestry. This narrow focus limits the generalizability and understanding of the causal relationship between metabolites and blood pressure in other ancestral populations. Thirdly, we used the summary data, which means we cannot explore any potential nonlinear effects or whether there might be differences in effect between different groups (such as male and female). To obtain a more comprehensive understanding, future investigations should include individuals from diverse ancestral backgrounds.

## Conclusions

Our findings identified 12 metabolites and 22 metabolites that had a causal effect on DBP and SBP respectively, providing important information for understanding the etiology and pathogenesis of hypertension.

### Electronic supplementary material

Below is the link to the electronic supplementary material.

Supplementary Material 1

## Data Availability

The summary statistics of SNP-metabolite associations from the CLSA study were available from the NHGRI-EBI GWAS Catalog (https://www.ebi.ac.uk/gwas/) with accession number GCST90199621-90201020. The summary statistics of SBP and DBP GWAS were available from the IEU OpenGWAS project (https://gwas.mrcieu.ac.uk/) with GWAS ID ieu-b-38 and ieu-b-39.
